# Feasibility of a Virtual Reality Intervention Protocol to Improve Cognitive and Behavioral Skills in Older Adults at Increased Risk of Developing Dementia

**DOI:** 10.2196/77111

**Published:** 2025-12-11

**Authors:** Oshadi Jayakody, Mirnova Ceïde, Joe Verghese, Robert Carrera, Hussain Doriwala, Sunil Agrawal, Judy Pa, Helena Blumen

**Affiliations:** 1 Department of Medicine Albert Einstein College of Medicine Bronx, NY United States; 2 Department of Psychiatry Albert Einstein College of Medicine Bronx, NY United States; 3 Department of Neurology Stony Brook Medicine Stony Brook, NY United States; 4 Department of Mechanical Engineering Columbia University New York, NY United States; 5 Department of Neurosciences University of California, San Diego San Diego, CA United States

**Keywords:** pilot study, community engagement studio, Alzheimer disease, clinical trials, skills training

## Abstract

This pilot study offers preliminary evidence that a virtual meal-preparation task is feasible for older adults and highlights that the community engagement studios are an effective approach to generate community-informed strategies to enhance intervention designs and reach.

## Introduction

Virtual reality (VR) offers immersive, interactive environments that simulate real-world tasks—providing opportunities for cognitive stimulation through increasing task complexity and real-time feedback. While prior studies report feasibility and cognitive benefits of VR interventions in older adults (ie, improved memory and attention [1-3]), transfer of benefits to real-world functioning is limited [4,5]. Additionally, despite facing disproportionately high dementia risk, older adults from minoritized backgrounds were underrepresented in prior interventions [6]. We are developing a VR intervention to promote cognitive and daily functioning in Black and Hispanic older adults. This study aimed to generate preliminary data to support the proposed large-scale feasibility study by testing the feasibility and safety of an ecologically valid virtual meal-preparing task (involving planning, memory, and visuospatial skills) and obtaining feedback from community experts to inform intervention refinement.

## Methods

### Overview

**Figure 1:**
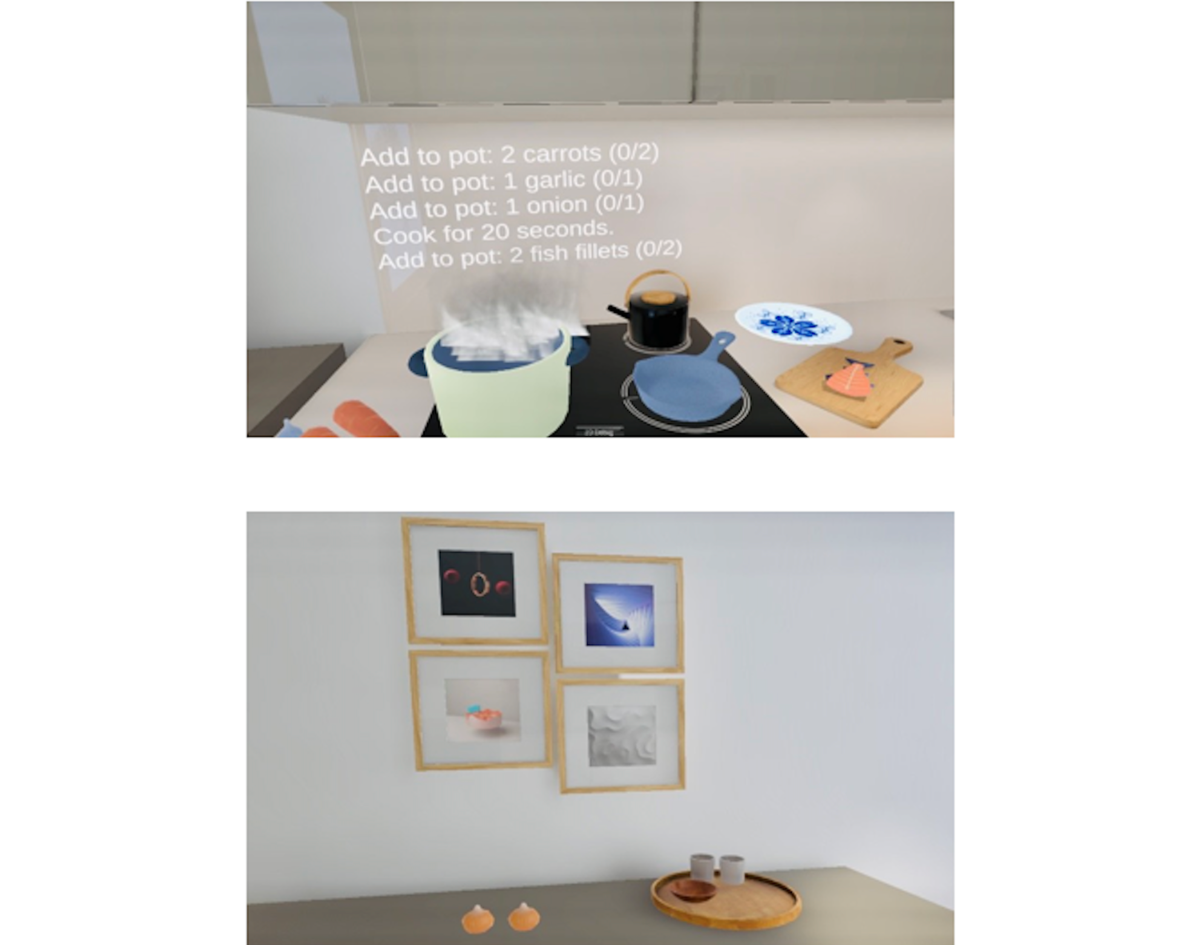
The virtual kitchen.

Eight adults aged ≥65 years at risk for dementia (AD8 Dementia Screening Interview score ≥1 [7]) and 8 young adults (aged 18-30 years) completed a single-session virtual meal-preparing task, developed in Unity 3D (2022 LTD version; Unity Technologies; Figure 1). Seated participants wore an HTC Vive headset and used wireless hand controllers. To assist navigation, 3D cues (eg, a stovetop) were placed. Personalized training sessions were provided to reduce usability barriers. The task involved 5 sequential steps engaging goal-setting, working memory, planning, visuospatial functions, and memory—mimicking a real-world kitchen. Task completion time, errors, motion sickness [8], simulator sickness [9], acceptability (Likert ratings on headset comfort and instruction clarity), and self-efficacy were compared across groups to understand older adults’ ability to engage in VR. Given the small sample, we report descriptive statistics to summarize outcomes.

A 1-hour community engagement studio (CES) was held at a community center with 16 older Black/Hispanic adults. The project lead hosted an information session to invite participants and delivered a 10-minute, jargon-free presentation outlining the design and goals of the proposed intervention. A bilingual facilitator guided a prestructured discussion to obtain feedback on the study’s significance, recruitment strategies, eligibility criteria, and duration. Responses were documented in real time and thematically coded by 2 investigators to identify key insights.

### Ethical Considerations

Ethics approval was obtained from the institutional review board of Albert Einstein College of Medicine, New York (2023-14856). Written informed consent was obtained from all participants prior to enrollment. Participant confidentiality was maintained through the use of deidentified data.

## Results

This study included 8 older adults (mean age 76.2, SD 7.9 years) and 8 young adults (mean age 25.0, SD 2.6 years), comprising, respectively, 5 (62%) and 4 (50%) women, 3 (38%) and 1 (12%) Hispanic individuals, and 2 (25%) and 1 (12%) non-Hispanic Black individuals. Older adults had a mean 12.8 (SD 8.3) years of education and young adults had 17.1 (SD 1.6) years. Older adults showed longer completion times (235.0, SD 99.6 vs 74.7, SD 18.9 seconds) and more errors (1.2, SD 1.3 vs 0.1, SD 0.3). Simulator sickness was minimal: pretest scores were 0.03 (SD 0.05) and 0.16 (SD 0.15); posttest scores were 0.02 (SD 0.03) and 0.07 (SD 0.06) for older and young adults. No safety concerns were reported except minor sweating due to the headset.

Acceptability scores (range 0-16) averaged 13.3 (SD 1.5) for older adults and 14.3 (SD 1.18) for young adults, and 6 (75%) older and 7 (88%) young adults reported enjoying the task. All participants expressed high self-efficacy for future VR use. The virtual environment was rated as realistic by 5 (62%) older adults; 2 (25%) somewhat agreed, and 1 (12%) disagreed.

Textbox 1 shows thematic insights from the CES. Community experts viewed the intervention as highly relevant. Health fairs and educational talks were identified as effective recruitment strategies. The bilingual flyer was well received, with comments to improve visual appeal. Eligibility for magnetic resonance imaging was considered a potential barrier. The proposed intervention duration was not seen as burdensome, though interventions conducted at community centers were preferred.

**A summary of key insights from the community engagement studio.**

**Significance of the intervention**

- Participants felt that the proposed intervention addresses an important question (improving cognitive and behavioral skills to delay the onset of Alzheimer disease and related dementias), considering their lived experience of family members and friends affected by Alzheimer disease and related dementias.

**Recruitment strategies and material**

- Suggested recruitment strategies included community outreach through educational talks.
- Magnetic resonance imaging at health fairs (which is an outcome assessment in the proposed intervention) might limit recruitment due to contraindications.
- Participants provided input to make the study flyer more appealing; they appreciated that the information is available in both English and Spanish.

**Study duration**

- Committing to the intervention duration (twice weekly for 4 weeks) was not considered burdensome.
- Suggestions to increase retention and adherence included providing free transportation, regular reminders, and refreshments.
- Most participants preferred that the intervention be conducted in a community setting rather than an academic study center.

**Use of technology and intervention appropriateness**

- Community experts expressed interest in using virtual reality and the use of an electronic pillbox (a suggested outcome assessment in the proposed intervention).
- The experts also agreed that the proposed virtual tasks were appropriate, culturally relevant, and reflected their daily activities.

## Discussion

### Principal Findings

This pilot study provides preliminary evidence supporting the feasibility of a virtual meal-preparing task in older adults. While older adults required more guidance, they completed the task in <4 minutes and reported high self-efficacy. The CES allowed us to obtain valuable, community-driven insights to improve recruitment, adherence, and delivery of the proposed intervention.

Prior VR interventions lasting 6 to 12 weeks (2-3 sessions/week; 30-45 minutes each), have shown cognitive benefits in older adults [2,3]. Our findings support progressing toward a similar structure, informed by user data and local community input. The brief training period was crucial in optimizing task completion and building confidence. The absence of simulator sickness—likely due to the seated setup and spatial cues—supports our safety approaches. Minor headset discomfort highlights the need for lighter headsets in future studies. CES insights reaffirm the value of involving community experts early in research development to enhance relevance and effective delivery.

Limitations include the small sample and single-session format, restricting statistical inference and long-term cognitive outcome assessments.

### Conclusions

These findings directly informed the next phase of our intervention while highlighting the promise of developing community-informed VR interventions for older adults.

## Abbreviations

CES: community engagement studio
VR: virtual reality

## Data Availability

Data from the pilot testing of the virtual task are available upon request from the corresponding author.
